# Trafficking Processes and Secretion Pathways Underlying the Formation of Plant Cuticles

**DOI:** 10.3389/fpls.2021.786874

**Published:** 2022-01-05

**Authors:** Glenn Philippe, Damien De Bellis, Jocelyn K. C. Rose, Christiane Nawrath

**Affiliations:** ^1^Plant Biology Section, School of Integrative Plant Science, Cornell University, Ithaca, NY, United States; ^2^Department of Plant Molecular Biology, University of Lausanne, Lausanne, Switzerland; ^3^Electron Microscopy Facility, University of Lausanne, Lausanne, Switzerland

**Keywords:** cutin, suberin, cuticle, cell wall, ABC-transporter, secretion, transport, extracellular vesiculo-tubular body

## Abstract

Cuticles are specialized cell wall structures that form at the surface of terrestrial plant organs. They are largely comprised lipidic compounds and are deposited in the apoplast, external to the polysaccharide-rich primary wall, creating a barrier to diffusion of water and solutes, as well as to environmental factors. The predominant cuticle component is cutin, a polyester that is assembled as a complex matrix, within and on the surface of which aliphatic and aromatic wax molecules accumulate, further modifying its properties. To reach the point of cuticle assembly the different acyl lipid-containing components are first exported from the cell across the plasma membrane and then traffic across the polysaccharide wall. The export of cutin precursors and waxes from the cell is known to involve plasma membrane-localized ATP-binding cassette (ABC) transporters; however, other secretion mechanisms may also contribute. Indeed, extracellular vesiculo-tubular structures have recently been reported in *Arabidopsis thaliana* (Arabidopsis) to be associated with the deposition of suberin, a polyester that is structurally closely related to cutin. Intriguingly, similar membranous structures have been observed in leaves and petals of Arabidopsis, although in lower numbers, but no close association with cutin formation has been identified. The possibility of multiple export mechanisms for cuticular components acting in parallel will be discussed, together with proposals for how cuticle precursors may traverse the polysaccharide cell wall before their assimilation into the cuticle macromolecular architecture.

## Introduction

**Figure 1:**
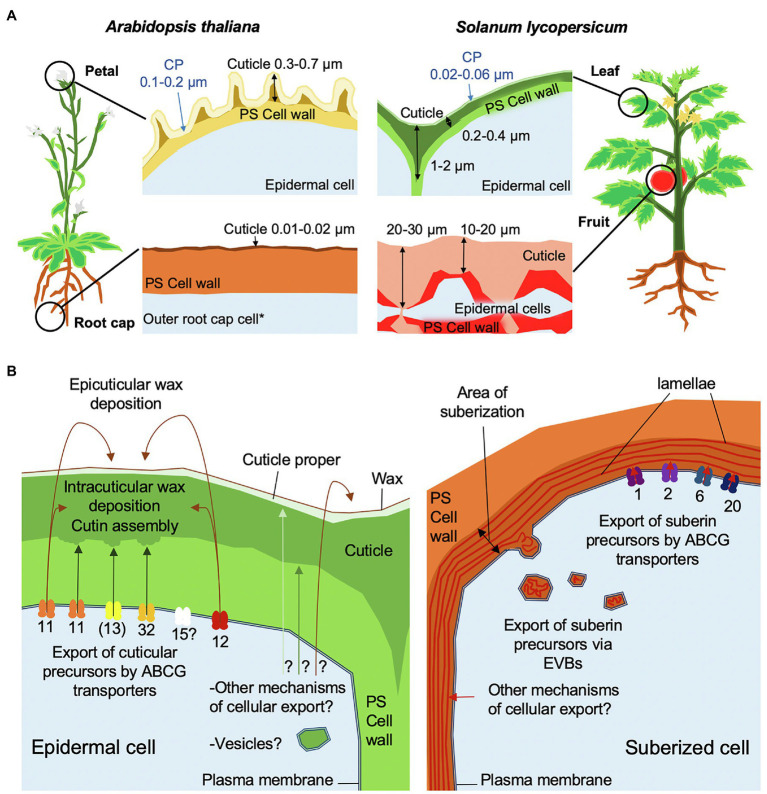
Schematic overview of cuticle assembly. **(A)** Morphologies of cuticles from different organs of tomato and Arabidopsis. Cuticle thickness measurements were based on TEM images from Martin et al. (2017), Mazurek et al. (2017), and Berhin et al. (2019). The cuticle proper (CP) is indicated by blue arrows. ^*^The root cap cuticle is only present on the first outer root cap layer formed during the development of a main root or lateral root. **(B)** Models of cuticle and suberin cellular export and trafficking mechanisms. ABCG transporters are annotated by their respective numbers in Arabidopsis. Brackets indicate that ABCG13 is only involved flowers. Arrows show the trafficking direction of lipidic components to their final destination. Question marks indicate hypothetical mechanisms. Note that LTPs have not been included in this model.

During plant organ development, a lipidic, hydrophobic cuticle is deposited on the nascent epidermal surface of the entire embryo (Ingram and Nawrath, 2017; Berhin et al., 2019), where it forms an intimate association with the underlying hydrated polysaccharide cell wall. Cuticle biosynthesis continues during organ expansion and is fine-tuned by developmental signals and environmental conditions to fulfill multiple roles. These include biomechanical support to maintain organ integrity, a barrier that limits the diffusion of a wide range of molecules between epidermal cells and the plant surface, and a layer that prevents organ fusion (Yeats and Rose, 2013; Ingram and Nawrath, 2017). The cuticle of the shoot of a seedling is maintained on the surface of organs in their primary growth stage during the entire life of the plant (Figure 1A), and cuticles can also line internal structures, such as the sub-stomatal chamber and locular cavity of some fruits. In contrast, the cuticle encasing the root of a seedling is shed with the embryonal root cap cell layer after seedling establishment (Figure 1A), at which time other extracellular protective structures, including mucilage sheaths, Casparian strips and suberin lamellae, have been formed in different cell types (Berhin et al., 2019).

The most abundant component of the cuticle is the polyester cutin, which is mainly composed of fatty acids C16–C18 in length carrying an oxygen-containing group (hydroxy, epoxy, or carboxy) in the ω or mid-chain positions of the acyl chain. In addition, relatively low amounts of glycerol and hydroxycinnamic acids are often detected as components of the polyester (Fich et al., 2016). Cuticular waxes, composed of very-long chain fatty acids (VLCFA) of C26–C34 and their derivatives (aldehydes, ketones, alcohols, alkanes, and wax esters), and alicyclic compounds (triterpenoids and flavonoids) impregnate the cutin matrix (intracuticular) or accumulate on the surface (epicuticular), where they may form films or crystal structures, depending on their amount and composition (Jetter et al., 2006; Jetter and Riederer, 2016).

Cutin is assembled in the apoplast from precursors that are generated within the cell (Fich et al., 2016; Philippe et al., 2020b). Hydroxylated fatty acids, synthesized by members of the cytochrome P450 families 77 and 86 are covalently linked to glycerol by glycerol 3-phosphate acyl transferase (GPAT) proteins at the endoplasmic reticulum (ER) membrane, resulting in monoacylglycerols (Gidda et al., 2009; Yang et al., 2012). Several acyl transferases of the BAHD family that are localized in the cytoplasm are also essential for the formation of the cutin polyester: for example, DEFECTIVE IN CUTICULAR RIDGES (DCR) is required for the incorporation of mid-chain oxygenated fatty acids into cutin (Panikashvili et al., 2009; Lashbrooke et al., 2016). In addition, DEFICIENT IN CUTIN FERULATE (DCF), a member of the BAHD family that has acyl-coenzyme A (CoA)-dependent acyl-transferase activity involving ferulic and sinapic acids, incorporates ferulate into cutin (Rautengarten et al., 2012). The formation of adducts with glycerol or other molecules may provide a mechanism to circumvent potential disturbance of membranes caused by free hydroxyacids (Douliez, 2004). Notably, all known cutin precursors are amphiphilic, reflecting the oxygenation of the component fatty acids and the conjugation to hydrophilic compounds (glycerol) or other amphiphilic compounds, such as hydroxycinnamic acids. Cuticular wax components are synthesized at the ER *via* the alkane-forming or alcohol-forming pathways after fatty acid elongation (Lewandowska et al., 2020). Wax molecules, particularly alkanes, are considerably more hydrophobic than cutin precursors.

While the core frameworks of the biosynthetic pathways of cutin and waxes have been generally defined, many questions remain regarding the export mechanisms of the cutin precursors and waxes; the trafficking processes across the hydrated primary cell wall to their point of assembly on the outer face of the wall, and factors that influence the subsequent assembly of the cutin scaffold and associated waxes.

In this article, we discuss the routes of export of cutin precursors and wax components across the plasma membrane (PM) and through the cell wall to their final destination in the cuticle. We compare these processes with the export of precursors of suberin, a polyester that is closely related to cutin, but that is often described as having longer monomer acyl chain lengths (C18–C28) than cutin and a higher content of phenolic compounds. Suberin is deposited in specialized cell types, or in response to tissue damage, often in the form of lamellae that are present between the PM and the bulk of the primary polysaccharide wall. This is a notable difference from cutin, which accumulates on the outer face of the primary wall. The mechanistic or structural basis for the differences in localization of the two polyester types has not yet been elucidated (Philippe et al., 2020b).

## Export of Cuticular Components by ABC Transporters

In organisms of all kingdoms ABC-transporters, consisting of nucleotide binding domains (NBD) and transmembrane domains (TMD), transport a broad range of molecules with different structure and properties across the PM. Some act as flippases, translocating acyl lipids from one leaflet of the membrane to the other (Lopez-Marques et al., 2015). Reaction mechanisms for binding different types of substrates and for the transport process (import/export/flippase) have been elucidated (Lopez-Marques et al., 2015; Lewinson et al., 2020). The different domains of ABC-transporters may be comprised of separate polypeptide chains, as is typical in bacteria. In eukaryotes, so-called ABC half-transporters, each of which is encoded by a single gene, are composed of one TMD and one NBD. These are thought to require dimerization to form a functional unit. Full ABC transporters, composed of two TMDs and NBDs, are formed by a single polypeptide chain. ABC-transporters in plants can be grouped into eight different families (ABCA–ABCI; ABCH is not found in plants). ABC-transporters of the G-family have the NBD at the N-terminus and the TMD at the C-terminus of the protein. In fungi and plants, in addition to ABCG half-transporters (previously called as WBC transporters), full ABCG transporters (termed Pleiotropic Drug Resistance (PDR)-type ABCG transporters) are also present (Verrier et al., 2008).

### ABCG Half-Transporters Are Required for Cuticle Formation

A clade of closely related ABCG half-transporters has been associated with the export of cuticular components (Do et al., 2018), which in Arabidopsis comprises four members: AtABCG11, AtABCG12, AtABCG13, and the currently uncharacterized AtABCG15 (Pighin et al., 2004; Bird et al., 2007; Luo et al., 2007; Ukitsu et al., 2007; Panikashvili et al., 2010, 2011; Figure 1B). Notably, a different clade of ABCG half-transporters (AtABCG1, AtABCG2, AtABCG6, and AtABCG20) is involved in the extracellular deposition of suberin (Yadav et al., 2014; Shanmugarajah et al., 2019; Figure 1B). AtABCG11 contributes to both wax and cutin export, while AtABCG12 is only required for wax export and AtABCG13 only for cutin precursor export (Pighin et al., 2004; Bird et al., 2007; Panikashvili et al., 2011).

Substrate specificities of ABCG-half transporters seem to be largely dependent on homodimer or heterodimer formation: for example, AtABCG11 forms homodimers and heterodimers with AtABCG12 *in vivo* (Bird, 2008; McFarlane et al., 2010). ABCG11 likely transports cutin precursors as a homodimer that forms with high affinity (Bird et al., 2007). The heterologous expression of AtABCG11 in protoplasts of *Nicotiana benthamiana* was observed to lead to the export of free and glycerol-bound hydroxylated fatty acids, consistent with a role in cutin precursor export (Elejalde-Palmett et al., 2021). Nevertheless, the possibility of heterodimerization with other ABCG half-transporters cannot be excluded. While *AtABCG11* is expressed in all Arabidopsis organs, *ABCG13* expression is restricted to inflorescences and specific positions in other organs. Whether this specific expression pattern relates to particular cutin precursor export capacities needs further investigation.

*PpABCG7*, a member of this clade of ABCG half-transporters, is required for wax export in the moss *Physcomitrium* (*Physcomitrella*) *patens* indicating that the role of this ABCG transporter clade has been conserved over at least 450 million years of plant evolution (Buda et al., 2013). ABCG function in the formation of impregnations of the cell wall with lipidic components may have arisen even earlier in various charophyte algal lineages (Kondo et al., 2016; Philippe et al., 2020b). Indeed, *ABCG11* homologues have been identified in extant charophyte algae, the sister lineage of embryophytes, and the size of the family has increased substantially during the emergence and evolution of land plants (Philippe et al., 2020b). This is consistent with the capacity to secrete and assemble hydrophobic cuticles being a prerequisite for plant colonization of truly terrestrial habitats.

### ABCG Full-Transporters Export Cutin Precursors

In addition to ABCG half-transporters, ABCG full-transporters play essential roles in cutin precursor export (Bessire et al., 2011; Chen et al., 2011b; Garroum et al., 2016; Figure 1B). The downregulation or knockout of *AtABCG32* homologs (named as *ABCG31* in monocots) in rice (*Oryza sativa*) and barley (*Hordeum vulgare*) results in severely impaired cuticle diffusion barrier properties (Chen et al., 2011a,b; Garroum et al., 2016). Additionally, the polysaccharide cell wall-cuticle interface is severely disrupted in the rice mutant (Garroum et al., 2016). In Arabidopsis, which has a cutin composition that is unusually rich in unsaturated dicarboxylic acids, ABCG32 plays a more pronounced role in cuticle formation of organs with a higher proportion of hydroxy acids (Bessire et al., 2011; Fabre et al., 2016). Although *AtABCG32* homologs have been tentatively identified in a few non-vascular plant lineages, it appears that at least one homolog is present in all seed plants (Philippe et al., 2020b). A duplication of *AtABCG32* has been identified in members of the Solanaceae (Elejalde-Palmett et al., 2021); however, whether the two paralogs have evolved different substrate specificities is still an open question. In the tobacco protoplast system, ABCG32 homologs transport oxygenated cutin precursors without selectivity for the structure at the terminal carbons or the mid-chain position, raising the possibility that these proteins may also transport yet uncharacterized cutin precursors (Elejalde-Palmett et al., 2021).

Full ABCG transporters are often expressed in the same organ and developmental stage as the ABCG half-transporters: for example, *AtABCG11*, *AtABCG13*, and *AtABCG32* are all expressed in floral organs. However, single *atabcg* mutants exhibit significant reductions in cutin levels, as well as other specific phenotypes, highlighting their non-redundant functions (Bird, 2008; Bessire et al., 2011; Panikashvili et al., 2011).

An important unanswered question is the mode by which the lipid-derived substrates interact with the ABCG transporters, i.e., as free molecules, as ligand-bound forms, or *via* the PM. This question is particular pertinent for the hydrophobic wax molecules. The export of the amphiphilic cutin precursors can be directly addressed with transport assays using radiolabeled precursors. However, the export of the more hydrophobic wax molecules has not yet been characterized since they partition into membranes *in vitro*. Consequently, there is not yet direct experimental evidence that the ABCG transporter formed by the AtABCG11/AtABCG12 heterodimer exports waxes.

## Understanding Cellular Trafficking of Cuticle Building Blocks and an Assessment of Alternative Export Pathways

A broad range of cell wall components, including structural proteins and cell wall matrix polysaccharides, such as hemicelluloses and pectins, are secreted *via* the canonical secretory pathway, in which vesicles derived from the trans-Golgi network fuse with the PM and deposit their cargo into the apoplast (Driouich et al., 2012). Lipophilic apoplastic components may follow similar transport pathways, and indeed early histological studies of the root endodermis revealed the presence of vesicles coincident with the deposition of suberin (Scott and Peterson, 1979). Consistent with this idea, the *atmin7* mutant, which is deficient in an ADP-ribosylation factor guanine exchange factor (ARF-GEF) protein, homologs of which function as regulators of the secretion pathway, exhibits reduced cutin deposition (Zhao et al., 2020). Similarly, the Arabidopsis *echidna* mutant, which has perturbed post trans-Golgi network (TGN) formation, shows decreased wax accumulation, although the presence of pleiotropic phenotypes complicates interpretation of these relationships (Gendre et al., 2013).

Besides export through the canonical secretion pathway, cell wall material may also be exported at direct contact sites between the cortical ER and the PM, independent of vesicular traffic (Samuels and McFarlane, 2012). In animal cells too, lipids traffic between the ER and other cell compartments *via* ER contact sites, and such a mechanism might also be used for the transport of cuticular lipids from the ER to the PM (Stefan et al., 2013; Wu et al., 2018).

Recently, a study of suberin formation in the root endodermis of Arabidopsis revealed that membrane-enclosed vesiculo-tubular structures (300–900 nm diameter), so called extracellular vesiculo-tubular bodies (EVBs), are tightly associated with the suberization process (Figure 1B; De Bellis et al., 2021). Notably, these EVBs are considerably larger in diameter than the vesicles of the canonical secretion pathway (30–100 nm). Remarkably, despite the resemblance of EVBs to multi-vesicular bodies (MVB), a specialized subset of endosomes that contain membrane-bound intraluminal vesicles, no evidence was found that EVBs are involved in recycling endosomes or are derived from Golgi vesicles, the trans-Golgi-network (TGN), or vacuoles (De Bellis et al., 2021). Moreover, cryo-fixation procedures reveal that the MVB-like appearance of these structures might be largely due to their swelling upon chemical fixation, and that their *in vivo* appearance is more lens-shaped, containing larger, less fragmented extracellular membrane tubules (De Bellis et al., personal communication). While blocking endosomal trafficking did not interfere with EVB- or suberin formation, blocking ER-to-*cis*-Golgi trafficking, as well as post-TGN secretory trafficking, affected both EVB accumulation and suberin formation, indicating that both early and later secretory pathway are required for EVB formation. The cargo of EVBs is hypothesized to be suberin precursors, since punctate structures of approximately 1 μm diameter, possibly corresponding to EVBs, were stained with the lipid dye fluorol yellow in early suberizing cells (De Bellis et al., 2021). In this context, it has been reported that free polyhydroxy acids form vesicles *in vitro* (Heredia-Guerrero et al., 2008), although it is not known whether suberin precursors, i.e., largely ω-hydroxy and dicarboxylic acids bound to glycerol, spontaneously form vesicles, nor whether such a process occurs *in vivo*. Recently, EVBs have also been reported in suberized bundle sheath cells in maize, further supporting the link between EVBs and suberin formation (Gao et al., 2021). In addition to suberin precursors, the EVB cargo may include enzymes catalyzing suberin formation, polymerization, or a broader collection of cell wall components.

An intriguing question is whether EVBs may also be associated with cutin formation. We addressed this using histological approaches to study tomato (*Solanum lycopersicum*) fruit and Arabidopsis petals, both of which have cutin that is rich in 10, 16 diOH C16:0 acids (Martin and Rose, 2014; Mazurek et al., 2017). In addition to wild-type (WT) Arabidopsis, we examined several mutants that have a reduction in cutin abundance (including in 10,16-diOH C16:0 levels) due to distinct changes in cutin precursor formation (*cyp77a6*, *gpat6*, *dcr* single and double mutants) or a deficiency in ABCG32 (*pec1*) expression (Bessire et al., 2011; Mazurek et al., 2017).

**Figure 2:**
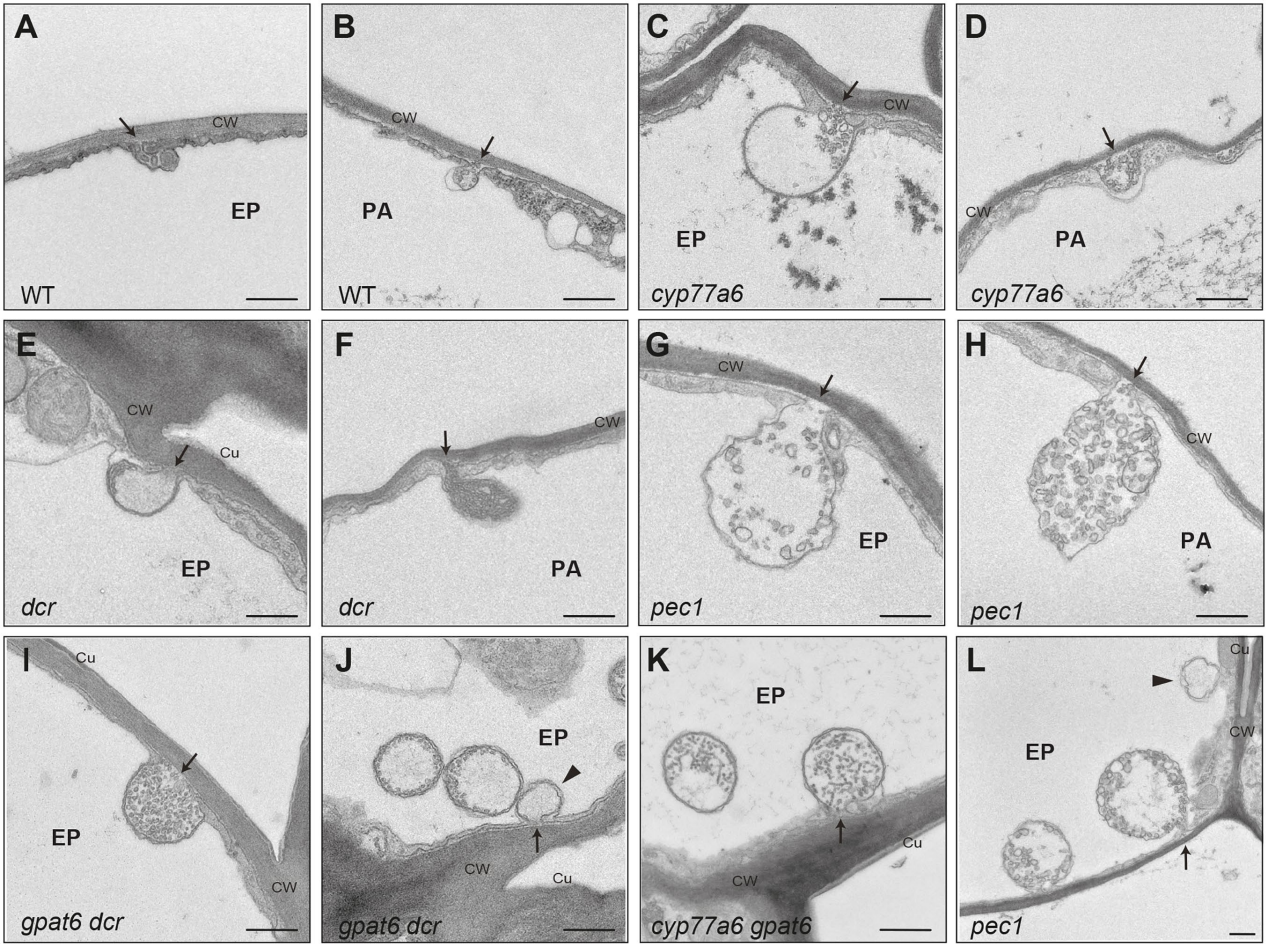
Extracellular vesiculo/tubular bodies in Arabidopsis. Transmission electron micrographs of extracellular vesiculo/tubular bodies (EVBs) visualized in Arabidopsis petals have connections to the cell wall. In addition, similar bodies were seen that were hypothesized to have the cell wall connection in a different plane of section. **(A,B)** Small EVBs in WT; **(C–L)** large EVBs in Arabidopsis cutin mutants. Arrows point to the connection to the cell wall; arrow head points to plasma filled bodies potentially having vesicles in another plane of section; Ep, epidermal cell; pa, parenchyme cell; CW, cell wall; Cu, cuticle. Scale bar represents 500 nm.

Notably, no EVB-like structures were observed during the expansion phase of WT tomato fruit development, at which point very large amounts of cutin are synthesized and deposited. In Arabidopsis, WT petals only very small EVB-like structures were present (Figure 2). However, large EVB-like structures (up to 2,500 nm in diameter), similar to these associated with suberization of Arabidopsis root tissues, were observed in all the investigated cutin-related mutants (Bessire et al., 2011; Mazurek et al., 2017). Interestingly, large EVBs were also seen in WT leaves, which have a cutin composition that it more similar to suberin than is cutin from petals (Nawrath et al., 2013). Nevertheless, in shoots, EVBs were not only specific to epidermal cells, but were also present at the periphery of internal cells, suggesting a role in the formation of multiple specialized cell wall types (Figure 2). Furthermore, EVBs in organs of the shoot were present in lower numbers (a maximum of 0.5 EVBs/cell section) than in suberizing root tissues (eight EVBs/cell section; De Bellis et al., 2021). In addition, the size and internal structure of EVBs varied considerably, not only between different genotypes but also between different preparations of sections from the same sample. This raises the question of whether these phenotypic characteristics are not only influenced by cellular metabolism and developmental trajectories, but also by the barrier properties of the cuticle affecting the chemical fixation and embedding procedure. Accordingly, the use of fixation methods that minimize the introduction of artefacts, such as cryo-fixation, will be important for further studies of EVBs in the formation of different cell wall types.

Our observations and published data suggest that EVBs are related to the deposition of suberized cell walls, rather than to the formation of cutin. However, the possibility that they also carry other cargo cannot be excluded (Figure 1B).

## The Mysterious Path Through the Cell Wall to the Site of Cuticle Assembly

The mechanism by which cutin precursors traffic from the point of their deposition into the apoplast and then across the highly hydrated primary cell wall to the site of cutin assembly remains a poorly understood aspect of cuticle formation. An earlier suggestion was that lipid-transfer proteins (LTPs), which are small (7–9 kDa) and often abundant extracellular proteins, function as carriers that mediate the transport of cuticular components across the wall (Sterk et al., 1991; Pyee et al., 1994; Hollenbach et al., 1997; Yeats and Rose, 2008; Salminen et al., 2016). Consistent with this model, LTPs have been found to accumulate at the plant surface (Pyee et al., 1994; Yeats et al., 2010). There is also evidence that LTPs play a role in the deposition of cuticular waxes, as evidenced by a reduction in the very long chain fatty acid (VLCFA) content in Arabidopsis LTP mutants (DeBono et al., 2009; Lee et al., 2009; Kim et al., 2012). However, the reported decrease in wax load in the mutants did not exceed 25% and there did not appear to be an effect on the composition or amount of cutin monomers. It is also notable that none of the many cuticle-related mutants that have been identified in a range of plant species to date has been attributed to a defect in a non-anchored LTP protein. Indeed, the energy cost of synthesizing proteins that would act as chaperones and transport cutin and waxes, in the absence of a recycling mechanism, suggests that such a process is unlikely. This would, presumably, particularly be the case with organs that deposit massive cuticles such as fleshy fruits. Thus, LTPs may instead have other roles such as antimicrobial defense and signaling (Bakan et al., 2006; Yeats and Rose, 2008; Salminen et al., 2016; Balmant et al., 2021).

Given the large amounts of material needed to assemble the thick cuticles of some organs, such as tomato and pepper fruit cuticles (>1 mg/cm^−2^ cutin monomers; Figure 1A; Graça et al., 2002), we suggest that the movement of cuticular lipids across the apoplast is more likely to be a passive process that avoids investment in metabolically expensive transport proteins (Fich et al., 2016; Philippe et al., 2020b). Such a mechanism would involve the diffusion of amphiphilic cutin precursors and hydrophobic waxes in the hydrophilic environment of the cell wall as a physicochemical phase-separation process. Bakan and Marion (2017) suggested that cutin precursors might aggregate in aqueous environments due to their chemical properties and may be stabilized as lyotropic structures through association with polysaccharides and non-polar waxes (Bakan and Marion, 2017). Indeed, the studies of the behavior of mixtures of cutin fatty acids with pectin *in vitro* have led to the observation of stable aggregation (Guzman-Puyol et al., 2015; Manrich et al., 2017). An early association of cutin precursors with waxes and polysaccharides is consistent with features of maturing cuticles, such as deposition of intracuticular waxes and the embedding of specific polysaccharides within the cuticle characterized by their hydrophobicity (Philippe et al., 2020a). While there is not currently direct evidence of phase separation of cuticle components *in muro*, transmission electron microscopy images of the outer epidermal wall and thick cuticles of tomato fruit often show patches of electron-dense material of increasing size nearer to the wall-cuticle interphase (Girard et al., 2012; Yeats et al., 2012) that are reminiscent of the coalescence of materials of differing hydrophobicity. It may be that a gradient of increasing hydrophobicity provides specific micro-/nano-scale environments for the aggregation of certain wall components, favoring the activities of specific enzymes that catalyze wall assembly. For example, tomato cutin synthase 1 (SlCUS1) was shown to localize in the cuticle and not the primary wall, indicative of the site of cutin polymerization (Girard et al., 2012; Yeats et al., 2012). Interestingly, over-expression of SlCUS1 using a constitutive promoter resulted in the appearance of polymeric cutin in non-epidermal wall layers, suggesting that the polymerizing activity is a limiting factor and that the cutin precursor substrate is mobile and not solely targeted to the organ surface (Yeats et al., 2012). The deposition of cutin in the anticlinal and sub-epidermal walls observed in some fruits suggests additional complexity in the modes and directionality of the secretion of cuticle components.

Phase separation of cuticle components may also result in compositional heterogeneity within a single cuticle. The cuticle is sometimes broadly described as a bi-layer, comprising an upper stratum, referred to as the ‘cuticle proper’, overlying the ‘cuticle layer’, which is thought to be less abundant in waxes but enriched with polysaccharides (Figure 1A; Yeats and Rose, 2013; Fernandez et al., 2016). While distinctly demarcated layers are not apparent in microscopic images of some cuticles, and a simple two-layer model may be overly simplistic, trafficking models should accommodate the formation of distinct zones (Figure 1B). For example, populations of epicuticular waxes are distinct from those of intracuticular waxes. However, it is not known whether cutin structure on the outer and inner face differ from each other, and new technologies are needed to resolve cuticle architecture at a higher degree of resolution. Regardless, phase separation of cuticular lipids with differing physicochemical properties seems a viable mechanism to both drive deposition of the cuticle and to establish higher order structures within the macromolecular cuticular matrix.

Another mystery is the basis of the different sites of deposition of the structurally related suberin and cutin polyesters, on the inner and outer faces of the polysaccharide wall, respectively (Philippe et al., 2020b). Suberin accumulates immediately after cell export and there is no evidence of diffusion of its precursors. An important factor in this regard may be that suberin has a relatively high phenolic content, which could affect the mobility of its precursors and promote physical associations with lignin, a phenolic polymer that is deposited in cell secondary walls close to the PM (Philippe et al., 2020b). Another feature that may influence the sites of cutinization or suberization is the potential involvement of multiple classes of proteins associated with their coordinated secretion at the PM. For example, it has been reported that membrane-anchored LTPs may be involved in suberin export or deposition, consistent with polymerization immediately after secretion (Deeken et al., 2016; Lee and Suh, 2018). Clearly, much remains to be learnt about this process, and the underlying mechanistic basis and potential differences between the deposition of canonical cutin and suberin, or other structural intermediates, represents an exciting area of future study.

## Conclusion

Key aspects of transport processes underlying the formation of plant extracellular lipid matrices are slowly coming into focus. This has been enabled by advances in high-resolution imaging, molecular probes and reverse genetic targeting of candidate genes, as well as mutant characterization. However, fundamental questions remain regarding the relationship between composition, architecture and assembly, and the molecular and physicochemical basis for the organization of the cuticle and the cell wall-cuticle continuum is still essentially a blank canvas.

## Materials and Methods

### Transmission Electron Microscopy

Transmission electron micrographs of *A. thaliana* (accession Col) petals were obtained as described previously (Fabre et al., 2016; Mazurek et al., 2017). Micrographs of the areas of interest were taken as tiled scans with a transmission electron microscope JEOL JEM-2100Plus (JEOL Ltd., Akishima, Tokyo, Japan) at an acceleration voltage of 80 kV with a TVIPS TemCamXF416 digital camera (TVIPS GmbH, Gauting, Germany) using the SerialEM software package (Mastronarde, 2005). Tiled scans were aligned with the software IMOD (Kremer et al., 1996).

## Data Availability Statement

The raw data supporting the conclusions of this article will be made available by the authors, without undue reservation.

## Author Contributions

DB did experiments. CN evaluated data. GP, JR, and CN wrote the article. All authors contributed to the article and approved the submitted version.

## Funding

This work was supported by the Swiss National Science Foundation, Switzerland (grant 31003A-170127 to CN). JR was supported by an award (59-8062-9-003P) from the Agricultural Research Service, and a grant (2020-03667) from the Agriculture and Food Research Initiative, United States Department of Agriculture.

## Conflict of Interest

The authors declare that the research was conducted in the absence of any commercial or financial relationships that could be construed as a potential conflict of interest.

The reviewer JP declared a past co-authorship with the author GP to the handling editor.

## Publisher’s Note

All claims expressed in this article are solely those of the authors and do not necessarily represent those of their affiliated organizations, or those of the publisher, the editors and the reviewers. Any product that may be evaluated in this article, or claim that may be made by its manufacturer, is not guaranteed or endorsed by the publisher.
